# Mutation analysis of the *CTNS* gene in Iranian patients with infantile nephropathic cystinosis: identification of two novel mutations

**DOI:** 10.1038/hgv.2017.38

**Published:** 2017-10-05

**Authors:** Forough Sadeghipour, Mitra Basiratnia, Ali Derakhshan, Majid Fardaei

**Affiliations:** 1 Department of Medical Genetics, Shiraz University of Medical Sciences, Shiraz, Iran; 2 Department of Pediatric Nephrology, Namazi Hospital, Shiraz University of Medical Sciences, Shiraz, Iran; 3 Transgenic Technology Research Center, Shiraz University of Medical Sciences, Shiraz, Iran

## Abstract

Nephropathic cystinosis is an inherited lysosomal transport disorder caused by mutations in the *CTNS* gene that encodes for a lysosomal membrane transporter, cystinosin. Dysfunction in this protein leads to cystine accumulation in the cells of different organs. The accumulation of cystine in the kidneys becomes apparent with renal tubular Fanconi syndrome between 6 and 12 months of age and leads to renal failure in the first decade of life. The aim of this study was to analyze the *CTNS* mutations in 20 Iranian patients, from 20 unrelated families, all of whom were afflicted with infantile nephropathic cystinosis. In these patients, seven different mutant alleles were found, including two new mutations, c.517T>C; p.Y173H and c.492_515del, that have not been previously reported. In addition, we observed that c.681G>A, the common Middle Eastern mutation, was the most common mutation in our patients. Moreover, a new minisatellite or variable number of tandem repeat marker (KX499495) was identified at the *CTNS* gene. Seven different alleles were found for this marker, and its allele frequency and heterozygosity degree were calculated in cystinosis patients and healthy individuals.

## Introduction

Cystinosis (OMIM 219800), the most common cause of renal Fanconi syndrome, is a lysosomal transport disorder with an autosomal recessive inheritance pattern, resulting from different mutations in the *CTNS* gene that is located on chromosome 17p13.^1,2^ The *CTNS* gene contains 12 exons with a coding region of 1,104 bp.^3^ The last 10 exons encode a lysosomal transmembrane protein with 367 amino acids called cystinosin. This protein consists of seven putative transmembrane domains (TM) and two lysosomal targeting motifs.^4,5^ Cystinosin dysfunction leads to deficient cystine transport and accumulation in cells of different organs, particularly in the kidney, cornea and thyroid.^3^

Cystinosis has a worldwide incidence between 1:100,000 and 1:200,000 live births;^6^ its frequency, to the best of our knowledge, has not been studied in the Iranian population. Infantile nephropathic cystinosis (OMIM 219800) is the most common and severe form of cystinosis, and it affects 95% of all patients. Without treatment, patients show growth retardation, proximal tubular Fanconi syndrome (polyuria, polydipsia, proteinuria, glucosuria, acidosis, dehydration, electrolyte imbalance, salt craving and tetany) at 6–12 months of age and renal failure at the end of the first decade of life.^3,7–9^ As a result of cystine crystal accumulation in the cornea, thyroid, muscle, central nervous system and pancreas, different nonrenal problems, including photophobia,^10^ hypothyroidism,^11^ vacuolar myopathy,^12^ encephalopathy^13^ and pancreatic exocrine and endocrine insufficiency,^14,15^ occur in patients. There are two other types of cystinosis, the nephropathic juvenile form (OMIM 219900) with milder renal symptoms in adolescence or early adulthood^9^ and the non-nephropathic adult form (OMIM 219750), the only manifestation of which is photophobia.^16^

More than 120 different mutations have been reported in the *CTNS* gene (www.hgmd.cf.ac.uk). Patients with the infantile form of cystinosis have two loss-of-function mutations that abolish the cysteine transport activity of cystinosin or alter its subcellular localization. In the two milder forms of cystinosis, affected individuals are homozygous or compound heterozygous for an allele with partial activity.^17–19^ The most common mutation in the northern European population is the 57-kb deletion, in which the first 9 exons, a part of exon 10 and the gene’s upstream sequence are removed.^20,21^ Among cystinosis molecular studies in the Middle East, c.681G>A; p.E227E is a common mutation that has been reported with different frequencies in different Middle Eastern regions.^22^

In this study, we analyzed mutations in 20 Iranian patients with infantile nephropathic cystinosis. In addition, we determined the allele frequency of the newly identified minisatellite marker at the *CTNS* gene in cystinosis patients and healthy individuals.

## Materials and methods

### Patients

Twenty unrelated Iranian patients, 15 males and 5 females, with infantile nephropathic cystinosis were included in this study. All patients were diagnosed based on clinical presentations focusing on failure to thrive, signs of renal Fanconi syndrome observed by the pediatric nephrology group in the Namazi pediatric center, and the presentation of corneal crystals observed via slit lamp examination performed by an experienced ophthalmologist. All the patients gave informed consent before undergoing a DNA test for the *CTNS* mutation analysis based on the requirements by the ethics committee of Shiraz University of Medical Sciences.

Whole blood samples (5 ml) from the patients and their families were collected in EDTA tubes. Genomic DNA was extracted from leukocytes by the salting-out technique.

### Mutation screening

Primers were designed for exons 3–12 and the exon–intron boundaries of the *CTNS* gene. PCR and direct sequencing of the amplified PCR products were carried out for all patients. The sequences of all primers are available upon request. To confirm the novel missense mutation (c.517T>C), an amplification-refractory mutation system PCR was performed for 50 healthy individuals (100 chromosomes).

### Analysis of a newly identified variable number of tandem repeat (VNTR) marker at the *CTNS* gene

A set of primers was designed to determine the allele frequency and heterozygosity degree of a new VNTR marker within the *CTNS* gene. The forward primer was labeled on the 5′ end with a fluorescent dye. PCR amplification was performed for 20 unrelated cystinosis patients and 50 healthy individuals (100 chromosomes). Capillary gel electrophoresis of the PCR products was carried out using an ABI 3,500×l genetic analyzer. Finally, data analysis was performed using Gene Mapper v4.1. (Thermo Fisher Scientific, Waltham, MA, USA).

### Statistical analysis

The allele frequency and heterozygosity degree of the new VNTR marker were calculated by the following formulas:

$$\mathrm{Allele}\;\mathrm{frequency}\;\left(P\right)=\frac{count\;of\;allele\;of\;interest}{count\;of\;all\;different\;observed\;alleles\;in\;the\;locus\;of\;interest}$$

Heterozygosity degree=1−(*p*_1_^2^+*p*_2_^2^+*p*_3_^2^+…+*p_n_*^2^).^23^

## Results

Among 20 Iranian families with infantile nephropathic cystinosis that participated in this study, 16 families had a consanguineous marriage. The earliest manifestations in all patients were failure to thrive, dehydration and vomiting between 4 and 24 months of age. These patients were diagnosed between 6 and 24 months of age. There were 11 patients with rickets, and 10 patients were diagnosed with hypothyroidism, for whom l-thyroxin replacement therapy had been considered. Patients 4, 5, 8, 16 and 18 underwent renal transplantation between 6 and 8 years of age. All patients, except patient 17, showed corneal crystals and photophobia (Table 1).

**Figure 1:**
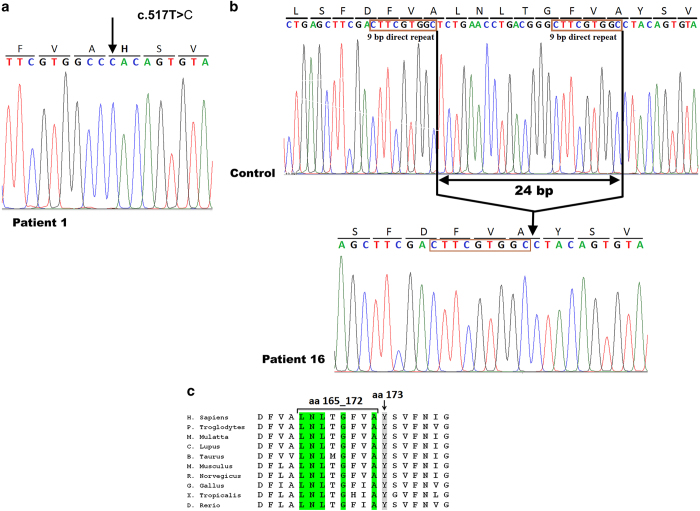
Sanger sequencing results of two new mutations in exon 8 of the *CTNS* gene. (**a**) In patient 1, a novel missense homozygous mutation was identified as c.517T>C; p.Y173H. (**b**) In patient 16, as well as patient 19 (not shown), a novel in-frame 24-bp deletion was identified as c.492_515del. The direct repeat sequences at the deletion breakpoints are shown. (**c**) Y173 and five of the eight removed amino acids (aa 165_172) in mutation c.492_515del were highly conserved among 10 different species.

Seven different mutations were detected in patients with nephropathic cystinosis (Table 2). Homozygosity was observed in 18 patients, and compound heterozygosity was observed in only two patients. Five of the seven mutations were reported in previous studies: NM_001031681.2(*CTNS*):c.613G>A; p.D205N missense mutation in two families, NM_001031681.2(*CTNS*):c.433C>T; p.Q145X nonsense mutation in one family, NM_001031681.2(*CTNS*):c.681G>A; p.E227E splice site mutation in 13 families, NM_001031681.2(*CTNS*):c.1015G>A; p.G339R missense mutation in two families and NM_001031681.2(*CTNS*):IVS11-12G>A, intronic splice site mutation in one family. We also found two new mutations, including one homozygous missense mutation (c.517T>C; p.Y173H) in one family (Figure 1a) and one homozygous 24 bp in-frame deletion (c.492_515del) that removed amino acids 165 to 172 (Figure 1b) and was observed in two unrelated families. These two mutations occurred in the second transmembrane (TM2) domain (PQ loop 1), and both of them contained highly conserved amino acids across species (Figure 1c). Mutation taster and mutation assessor tools were used for both novel mutations to confirm their pathogenic natures. Furthermore, a control group consisting of 50 healthy individuals (100 chromosomes) was also screened with the amplification-refractory mutation system PCR technique for novel missense mutations to ensure that this mutation did not exist in the normal population.

**Figure 2:**
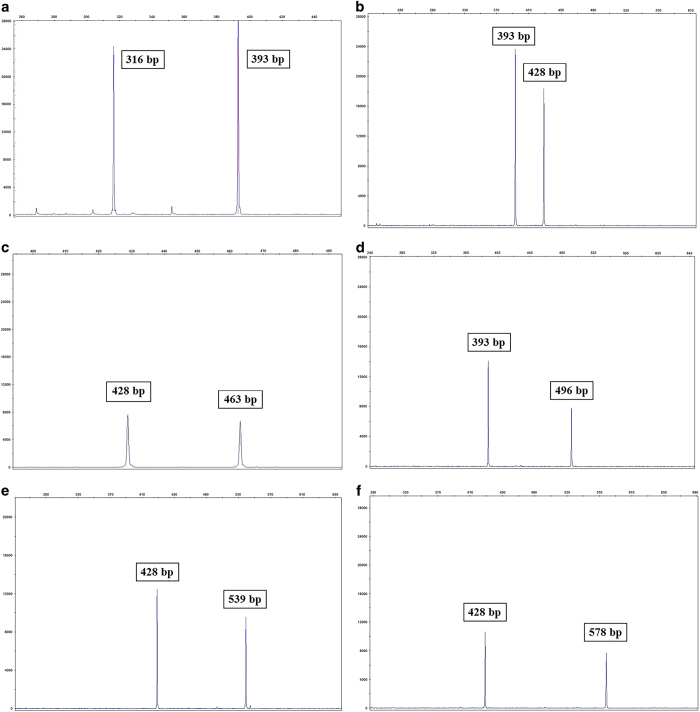
Electropherograms of seven different VNTR alleles in the *CTNS* gene have been observed in both the patient and control groups. Just three alleles with 393 bp (9 repeat units), 428 bp (10 repeat units) and 496 bp (12 repeat units) lengths were observed in the patient group (**b** and **d**). All seven VNTR alleles were found in the control group (**a**, **c**, **e** and **f**).

In this study, using the Tandem Repeat Finder tool (https://tandem.bu.edu/trf/trf.html), we identified two flanking consensus sequences in intron 7 of the *CTNS* gene. This C-rich region, with ~36 bp repeat units, constitutes a VNTR marker (GenBank accession number: KX499495) (https://www.ncbi.nlm.nih.gov/nuccore/kx499495). Seven different alleles were found for this minisatellite marker, which was analyzed using a genetic analyzer (Figure 2). Then, allele frequencies were calculated in both patient and control groups (Table 3). The observed heterozygosity for this marker was 56% in the normal population.

## Discussion

**Figure 3:**
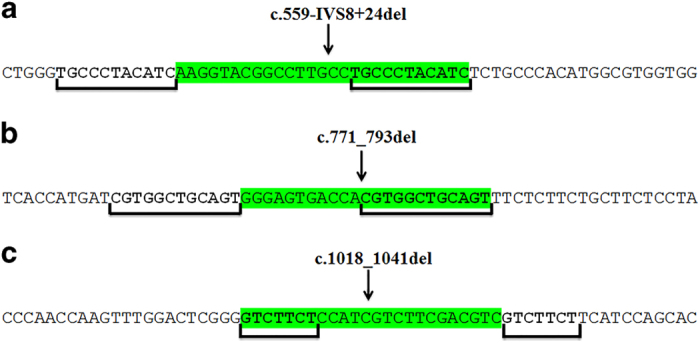
Three previously reported deletions (**a**, **b** and **c**) within the *CTNS* gene and presence of direct repeats around each deletion breakpoint.

To the best of our knowledge, reports proving the 57-kb deletion mutation has not been observed so far in any Middle Eastern studies, including Egypt,^22^ Iran,^24^ Turkey^25^ and Saudi Arabia,^26^ which supports the theory that this mutation is a founder mutation and is restricted to American/European populations.^22^ Therefore, in this study, direct sequencing of coding regions and exon–intron boundaries of the *CTNS* gene was performed instead of primary screening for a 57-kb deletion to find the mutations that caused the disease. An analysis of *CTNS* gene coding exons in 20 unrelated Iranian cystinosis patients revealed seven different mutations, of which 2 mutations have not been previously reported. The first novel mutation found in this study is the missense mutation (c.517T>C; p.Y173H) that was identified in patient 1 (Figure 1a). This mutation changed the highly conserved tyrosine at position 173 at the TM2 domain (exon 8) of the protein to the basic amino acid histidine. The second novel mutation consisted of the in-frame deletion (c.492_515del) in exon 8 that was identified in two unrelated families (P16 and P19) and removed 8 of the 21 amino acids from the TM2 domain (aa 165_172). Sequence analysis of the DNA surrounding deletion breakpoints revealed the presence of a 9-bp direct repeat (CTTCGTGGC) at both sides of the breakpoints (Figure 1b). Furthermore, we analyzed the sequences around the breakpoints of the three previously reported deletions in the *CTNS* gene; a 27 bp deletion (c.559-IVS8+24) in exon 8,^17^ a 23 bp deletion (c.771_793del) in exon 10^ref. ^^17^ and a 24 bp deletion (c.1018_1041del) in exon 12^ref. ^^26^ were surrounded by 11, 12 and 7 bp direct repeats, respectively (Figure 3). Misalignment of these short direct repeats provokes slipped-strand mispairing during DNA replication, which has also been previously reported for deletions in microorganisms,^27,28^ human β-globin and retinoblastoma genes.^29^ At the replication fork, after synthesis of the first direct repeat in the primer strand, dissociation of this repeat from the template strand and forward slip of the primer strand permit mispairing between two direct repeats. Continuing DNA replication leads to the deletion of one copy of the repeat and sequence lying between two direct repeats (Figure 1b).^30,31^

The previously reported splice site mutation, c.681G>A, that comprises 60% (or 24) of the mutant alleles of all patients in this study, is a common mutation in the Middle East and is the most common mutation in Iran on the basis of this study and a previous study of cystinosis in southwestern Iran.^24^ Eleven patients were homozygous and two patients were compound heterozygous for this mutation. According to former studies performed in the Middle East, this mutation is distributed with different frequencies in Saudi Arabia,^26^ Iran,^24^ Egypt^22^ and Turkey,^25^ but it has not been reported in any of the European and American populations until now. Thus, it can be called a founder mutation in the Middle East, with the highest frequency in Iran.

Other previously reported mutations were identified in our study that affect the transport activity of cystinosin, including the two missense mutations c.613G>A; D205N^32^ in the first inter-TM loop (exon 9) in patient 2 (in a homozygous state) and patient 17 (in a heterozygous state) and c.1015G>A; G339R^32^ in TM7 (exon 12) in patients 8 and 13 in homozygous states.^33^ The nonsense mutation, c.433C>T; Q145X in exon 7 leading to a truncated protein, was previously reported in Russia.^34^ This mutation was detected in a homozygous state in patient 3. IVS11-12G>A, a heterozygote intronic splice site mutation, was detected in patient 11. This mutation in intron 11 leads to a 323-residue truncated protein.^17^

In the present study, we identified a new minisatellite marker within the *CTNS* gene. This C-rich marker was located in intron 7 of the gene. Analysis of capillary electrophoresis data from 20 unrelated cystinosis patients revealed only three alleles with 9, 10 and 12 repeat units, with the highest frequency related to the allele having 10 repeat copies (75%). A capillary electrophoresis analysis of 100 chromosomes in the normal population in the Fars province of Iran revealed seven alleles with 7, 9, 10, 11, 12, 13 and 14 repeat copies. In the normal population, just as in the patient group, an allele with 10 repeat units had the highest frequency. Observation of only three of the seven alleles in the patient group may imply that these are disease-associated alleles for cystinosis. Furthermore, in all 13 patients with the mutation of c.681G>A, the mutation was linked to the 10 repeats allele of VNTR. Patients with a homozygous mutant allele were homozygous for this VNTR allele and patients with a heterozygous mutant allele were heterozygous for it. Most likely, these findings indicate that there is a common ancestor and the same origin for this common mutation and prove that c.681G>A is a founder mutation in the Middle East.

Nevertheless, to demonstrate this claim, analysis of this VNTR marker and several polymorphic STR markers in the vicinity of the *CTNS* gene is required in patients carrying this mutation in Iran and other Middle Eastern countries. Analysis of VNTR alleles in other patients showed that the novel mutation c.492_515del in P16 and P19 was associated with the VNTR allele with 9 repeat units and the G339R mutation in P8 and P13 was associated with the 10 repeat units allele, but the D205N mutation in P2 and P17 showed two different VNTR alleles, indicating two different origins for this mutation. Three remaining mutations, including novel missense mutations Y173H, Q145X and IVS11-12G>A, were associated with 9, 10 and 12 repeat units alleles, respectively.

Because of the high rates of consanguineous marriage in Iran, cystinosis, like many other autosomal recessive disorders, has a high frequency, especially in southwestern Iran. In our study, we found two novel mutations including c.517T>C; p.Y173H and c.492_515del. Since c.681G>A; p.E227E comprises the most mutant alleles in this (60%) study and in a previous (39.5%)^24^ study conducted in Iran, it can be considered the most common mutation in Iran. To quickly identify heterozygous carriers, primary screening for c.681G>A in at-risk Iranian individuals would be beneficial. The heterozygosity degree calculated for the newly identified marker in this study (56%) revealed that it is a relatively informative marker that can be used in carrier detection and as a confirming test for prenatal diagnosis of cystinosis. However, for more accurate determination of the heterozygosity degree of this marker, further extensive study of the marker in a large population is required.

## Additional information

**Publisher’s note:** Springer Nature remains neutral with regard to jurisdictional claims in published maps and institutional affiliations.

## Figures and Tables

**Table 1:** Clinical findings of patients with nephropathic cystinosis

*Patient /sex*	*Age in years*	*Consanguineous marriage*	*Onset age in months*	*Age at diagnosis in months*	*Fanconi syndrome*	*Rickets*	*Hypothyroidism*	*Corneal crystals*	*Renal transplantation*
P1/M	12	+	5	6	+	−	−	+	−
P2/M	9	+	18	18	+	+	+	+	−
P3/M	7	+	12	13	+	+	−	+	−
P4/M	10	+	10	11	+	+	−	+	+ (7 years)
P5/F	16	+	7	8	+	+	+	+	+ (8 years)
P6/F	6	+	8	10	+	−	+	+	−
P7/M	5	+	9	18	+	+	-	+	−
P8/M	12	+	12	13	+	−	+	+	+ (8 years)
P9/F	11	+	6	22	+	+	+	+	−
P10/M	4	+	6	7	+	−	+	+	−
P11/M	10	−	6	16	+	−	+	+	−
P12/M	5	+	4	9	+	+	−	+	−
P13/M	6	+	6	8	+	+	−	+	−
P14/M	8	+	8	22	+	+	−	+	−
P15/F	9	+	4	12	+	+	+	+	−
P16/M	18	+	5	25	+	−	+	+	+ (6 years)
P17/M	11	−	24	24	+	−	−	−	−
P18/M	10	−	12	24	+	−	+	+	+ (6 years)
P19/M	9	+	10	18	+	−	−	+	−
P20/F	4	−	11	13	+	+	−	+	−

**Table 2:** Genotypes of patients with infantile nephropathic cystinosis

*Patient ID*	*Mutant alleles NM_001031681.2(CTNS)*	*Location*	*Amino acid change*	*Protein effect*	*Reference*
P1	c.517T>C/c.517T>C	Exon 8	Y173H	aa^a^ change at TM2	This study
P2	c.613G>A/c.613G>A	Exon 9	D205N	aa change at 1st inter-TM loop	(Shotelersuk *et al.*^32^)
P3	c.433C>T/c.433C>T	Exon 7	Q145X	Truncated protein at AA145	(Kartamysheva *et al.*^34^)
P4	c.681G>A/c.681G>A	Exon 9	E227E	Alternative splicing	(Aldahmesh *et al.*^26^)
P5	c.681G>A/c.681G>A	Exon 9	E227E	Alternative splicing	(Aldahmesh *et al.*^26^)
P6	c.681G>A/c.681G>A	Exon 9	E227E	Alternative splicing	(Aldahmesh *et al.*^26^)
P7	c.681G>A/c.681G>A	Exon 9	E227E	Alternative splicing	(Aldahmesh *et al.*^26^)
P8	c.1015G>A/c.1015G>A	Exon 12	G339R	aa change at TM7	(Shotelersuk *et al.*^32^)
P9	c.681G>A/c.681G>A	Exon 9	E227E	Alternative splicing	(Aldahmesh *et al.*^26^)
P10	c.681G>A/c.681G>A	Exon 9	E227E	Alternative splicing	(Aldahmesh *et al.*^26^)
P11	c.681G>A/IVS11-12G>A	Exon 9/Intron 11	E227E/Frameshift	Alternative splicing/Truncated protein	(Aldahmesh *et al.*^26^)/(Attard 1999)
P12	c.681G>A/c.681G>A	Exon 9	E227E	Alternative splicing	(Aldahmesh *et al.*^26^)
P13	c.1015G>A/c.1015G>A	Exon 12	G339R	aa change at TM7	(Shotelersuk *et al.*^32^)
P14	c.681G>A/c.681G>A	Exon 9	E227E	Alternative splicing	(Aldahmesh *et al.*^26^)
P15	c.681G>A/c.681G>A	Exon 9	E227E	Alternative splicing	(Aldahmesh *et al.*^26^)
P16	c.492_515del/c.492_515del	Exon 8	aa 165-172del	Disruption of PQ loop 1 in TM2	This study
P17	c.613G>A/c.681G>A	Exon 9/Exon 9	D205N/E227E	aa change at 1st inter-TM loop /Alternative splicing	(Shotelersuk *et al.*^32^)/(Aldahmesh *et al.*^26^)
P18	c.681G>A/c.681G>A	Exon 9	E227E	Alternative splicing	(Aldahmesh *et al.*^26^)
P19	c.492_515del/c.492_515del	Exon 8	aa 165-172del	Disruption of PQ loop 1 in TM2	This study
P20	c.681G>A/c.681G>A	Exon 9	E227E	Alternative splicing	(Aldahmesh *et al.*^26^)

a Amino acid.

**Table 3:** VNTR alleles in the *CTNS* gene in patients with cystinosis and the control group

*Allele size (bp)*	*Number of repeats*	*Allele frequency (%) in the patient group*	*Allele frequency (%) in the control group*
316	7	0	1
393	9	20	24
428	10	75	65
463	11	0	1
496	12	5	6
539	13	0	2
578	14	0	1

## References

[bib1] Town M, Jean G, Cherqui S, Attard M, Forestier L, Whitmore SA et al. A novel gene encoding an integral membrane protein is mutated in nephropathic cystinosis. Nat Genet 1998; 18: 319. 9537412 10.1038/ng0498-319

[bib2] McDowell GA, Gahl WA, Stephenson LA, Schneider JA, Weissenbach J, Polymeropoulos MH et al. Linkage of the gene for cystinosis to markers on the short arm of chromosome 17. Nat Genet 1995; 10: 246–248. 7663525 10.1038/ng0695-246

[bib3] Nesterova G, Gahl WA. Cystinosis: the evolution of a treatable disease. Pediatr Nephrol 2013; 28: 51–59. 22903658 10.1007/s00467-012-2242-5 PMC3505515

[bib4] Kalatzis V, Cherqui S, Antignac C, Gasnier B. Cystinosin, the protein defective in cystinosis, is a H+-driven lysosomal cystine transporter. EMBO J 2001; 20: 5940–5949. 11689434 10.1093/emboj/20.21.5940 PMC125690

[bib5] Kalatzis V, Antignac C. New aspects of the pathogenesis of cystinosis. Pediatr Nephrol 2003; 18: 207–215. 12644911 10.1007/s00467-003-1077-5

[bib6] Gahl WA, Thoene JG, Schneider JA. Cystinosis. N Engl J Med 2002; 347: 111–121. 12110740 10.1056/NEJMra020552

[bib7] Wilmer MJ, Schoeber JP, van den Heuvel LP, Levtchenko EN. Cystinosis: practical tools for diagnosis and treatment. Pediatr Nephrol 2011; 26: 205–215. 20734088 10.1007/s00467-010-1627-6 PMC3016220

[bib8] Gahl W, Schneider J, Aula P.Lysosomal transport disorders: cystinosis and sialic acid storage disorders. The metabolic and molecular bases of inherited disease1995; 3: 3780.

[bib9] Gahl WA, Nesterova G. Cystinosis and its renal complications in children. Pediatr Nephrol 2014, 1–28.

[bib10] Gahl WA, Kuehl EM, Iwata F, Lindblad A, Kaiser-Kupfer MI. Corneal crystals in nephropathic cystinosis: natural history and treatment with cysteamine eyedrops. Mol Genet Metab 2000; 71: 100–120. 11001803 10.1006/mgme.2000.3062

[bib11] Chan AM, Lynch MJG, Bailey JD, Ezrin C, Fraser D. Hypothyroidism in cystinosis: a clinical, endocrinologic and histologic study involving sixteen patients with cystinosis. Am J Med 1970; 48: 678–692. 5420554 10.1016/s0002-9343(70)80002-x

[bib12] Gahl WA, Dalakas MC, Charnas L, Chen KTK, Pezeshkpour GH, Kuwabara T et al. Myopathy and cystine storage in muscles in a patient with nephropathic cystinosis. N Engl J Med 1988; 319: 1461–1464. 3185663 10.1056/NEJM198812013192206

[bib13] Broyer M, Tete MJ, Guest G, Bertheleme JP, Labrousse F, Poisson M. Clinical polymorphism of cystinosis encephalopathy. Results of treatment with cysteamine. J Inherit Metab Dis 1996; 19: 65–75. 8830179 10.1007/BF01799350

[bib14] Fivush B, Flick JA, Gahl WA. Pancreatic exocrine insufficiency in a patient with nephropathic cystinosis. J Pediatr 1988; 112: 49–51. 3335962 10.1016/s0022-3476(88)80119-7

[bib15] Fivush B, Green OC, Porter OC, Balfe JW, O'Regan S, Gahl WA. Pancreatic endocrine insufficiency in posttransplant cystinosis. Am J Dis Child 1987; 141: 1087–1089. 3307383 10.1001/archpedi.1987.04460100065027

[bib16] Gahl WA, Thoene J, Schneider J In: Scriver C, Beaudet A, Sly W et al. (eds). The metabolic and molecular bases of inherited disease. McGraw-Hill: New York, 2001.

[bib17] Attard M, Jean G, Forestier L, Cherqui S, van't Hoff W, Broyer M et al. Severity of phenotype in cystinosis varies with mutations in the CTNS gene: predicted effect on the model of cystinosin. Hum Mol Genet 1999; 8: 2507–2514. 10556299 10.1093/hmg/8.13.2507

[bib18] Thoene J, Lemons R, Anikster Y, Mullet J, Paelicke K, Lucero C et al. Mutations of CTNS causing intermediate cystinosis. Mol Genet Metab 1999; 67: 283–293. 10444339 10.1006/mgme.1999.2876

[bib19] Anikster Y, Lucero C, Guo J, Huizing M, Shotelersuk V, Bernardini I et al. Ocular nonnephropathic cystinosis: clinical, biochemical, and molecular correlations. Pediatr Res 2000; 47: 17. 10625078 10.1203/00006450-200001000-00007

[bib20] Anikster Y, Lucero C, Touchman JW, Huizing M, McDowell G, Shotelersuk V et al. Identification and detection of the common 65-kb deletion breakpoint in the nephropathic cystinosis gene (CTNS). Mol Genet Metabol 1999; 66: 111–116. 10.1006/mgme.1998.2790 10068513

[bib21] Touchman JW, Anikster Y, Dietrich NL, Braden Maduro VV, McDowell G, Shotelersuk V et al. The genomic region encompassing the nephropathic cystinosis gene (CTNS): complete sequencing of a 200-kb segment and discovery of a novel gene within the common cystinosis-causing deletion. Genome Res 2000; 10: 165–173. 10673275 10.1101/gr.10.2.165 PMC310836

[bib22] Soliman NA, Elmonem MA, van den Heuvel L, Abdel Hamid RH, Gamal M, Bongaers I et al. Mutational spectrum of the CTNS gene in Egyptian patients with nephropathic cystinosis. in JIMD Reports Springer, Berlin Heidelberg, 2014; 14: 87–97. 10.1007/8904_2013_288 PMC4213330 24464559

[bib23] Strachan T, Read AR. Genetic Mapping of Mendelian Characters. In: Human Molecular Genetics, 4th edn, Garland Science: New York. 2011, 449.

[bib24] Shahkarami S, Galehdari H, Ahmadzadeh A, Babaahmadi M, Pedram M. The first Molecular genetics analysis of individuals suffering from nephropatic cystinosis in the Southwestern Iran. Nefrologia 2013; 33: 308–315. 23640116 10.3265/Nefrologia.pre2012.Sep.11558

[bib25] Topaloglu R, Vilboux T, Coskun T, Ozaltin F, Tinloy B, Gunay-Aygun M et al. Genetic basis of cystinosis in Turkish patients: a single-center experience. Pediatr Nephrol 2012; 27: 115–121. 21786142 10.1007/s00467-011-1942-6 PMC3501933

[bib26] Aldahmesh MA, Humeidan A, Almojalli HA, Khan AO, Rajab M, AL-Abbad AA et al. Characterization of CTNS mutations in Arab patients with cystinosis. Ophthal Genet 2009; 30: 185–189. 10.3109/13816810903200953 19852576

[bib27] Viguera E, Canceill D, Ehrlich SD. In vitro replication slippage by DNA polymerases from thermophilic organisms. J Mol Biol 2001; 312: 323–333. 11554789 10.1006/jmbi.2001.4943

[bib28] Viguera E, Canceill D, Ehrlich SD. Replication slippage involves DNA polymerase pausing and dissociation. EMBO J 2001; 20: 2587–2595. 11350948 10.1093/emboj/20.10.2587 PMC125466

[bib29] Efstratiadis A, Posakony JW, Maniatis T, Lawn RM, O'Connell C, Spritz RA et al. The structure and evolution of the human β-globin gene family. Cell 1980; 21: 653–668. 6985477 10.1016/0092-8674(80)90429-8

[bib30] Levinson G, Gutman GA. Slipped-strand mispairing: a major mechanism for DNA sequence evolution. Mol Biol Evol 1987; 4: 203–221. 3328815 10.1093/oxfordjournals.molbev.a040442

[bib31] Ball EV, Stenson PD, Abeysinghe SS, Krawczak M, Cooper DN, Chuzhanova NA. Microdeletions and microinsertions causing human genetic disease: common mechanisms of mutagenesis and the role of local DNA sequence complexity. Hum Mutat 2005; 26: 205–213. 16086312 10.1002/humu.20212

[bib32] Shotelersuk V, Larson D, Anikster Y, McDowell G, Lemons R, Bernardini I et al. CTNS mutations in an American-based population of cystinosis patients. Am J Hum Genet 1998; 63: 1352–1362. 9792862 10.1086/302118 PMC1377545

[bib33] Kalatzis V, Nevo N, Cherqui S, Gasnier B, Antignac C. Molecular pathogenesis of cystinosis: effect of CTNS mutations on the transport activity and subcellular localization of cystinosin. Hum Mol Genet 2004; 13: 1361–1371. 15128704 10.1093/hmg/ddh152

[bib34] Kartamysheva N, Karagulyan NA, Tsygina YE, Sevostyanov KV, Pushkov AA, Mazo AM et al. Clinical, molecular-genetic characteristics of cystinosis in children of Russian Federation and perspectives of its treatment. Pediatriya named after GN Speransky 2014; 93: 39–42.

